# Exploring the role of mitochondrial metabolism and immune infiltration in myocardial infarction: novel insights from bioinformatics and experimental validation

**DOI:** 10.3389/fimmu.2025.1543584

**Published:** 2025-05-27

**Authors:** Jingyi Hou, Lihan Wang, Naiqiang Zhu, Xueli Li

**Affiliations:** ^1^ Beijing Key Laboratory of Traditional Chinese Medicine Basic Research on Prevention and Treatment for Major Diseases, Experimental Research Center, China Academy of Chinese Medical Sciences, Beijing, China; ^2^ Postdoctoral Mobile Research Station of China Academy of Chinese Medicine Sciences, Beijing, China; ^3^ Chengde Medical University, Chengde, China

**Keywords:** myocardial infarction, mitochondrial metabolism, immune infiltration, immunometabolism, bioinformatics analysis, differentially expressed genes, immunohistochemistry, ischemia

## Abstract

**Background:**

Mitochondrial metabolism and immune inflammation play pivotal roles in the MI pathogenesis of myocardial infarction (MI); however, their interplay remains unclear. This study aimed to clarify the roles of mitochondrial metabolism and immune infiltration in MI, using a combination of bioinformatics analyses and experimental validation.

**Methods:**

MI chip data (GSE96561, GSE181872, and GSE183272) were obtained from the Gene Expression Omnibus (GEO) database, and mitochondrial gene data were sourced from the MitoCarta3.0 database. Differentially expressed genes (DEGs) were identified and subjected to functional enrichment analyses. Mitochondria-related DEGs (mitoDEGs) were determined by intersecting DEGs with mitochondrial genes and associated Gene Ontology (GO) terms were analyzed using the Metascape database. A protein-protein interaction (PPI) network of mitoDEGs was constructed, and hub mitoDEGs associated with MI were identified using CytoHubba and molecular complex detection (MCODE) algorithms. Transcription factor (TF) and microRNA (miRNA) targets of hub mitoDEGs were predicted using iRegulon and miRWalk plug-ins, respectively, and a regulatory network involving TFs, hub mitoDEGs, and miRNA was established. Immune infiltration in MI was analyzed using ImmuCellAI, and the relationship between hub mitoDEGs and immune infiltration abundance was assessed using the Spearman method. Experimental validation of hub mitoDEGs, immune cell markers (F4/80, CD163 and CD86), and apoptosis-related proteins (BAX/BCL-2 and cleaved caspase-3) was conducted in MI mice, and the association with cardiac function was explored.

**Results:**

MitoDEGs in the MI group were significantly enriched in pathways related to mitochondrial transport and gene expression. Nine hub mitoDEGs closely associated with MI were identified. Immune analysis revealed increased infiltration of mast and plasma cells infiltration and decreased CD4 T cell infiltration in the MI immune microenvironment. Spearman analysis showed positive correlations between hub mitoDEGs and M1 macrophages, Th2 Cells, and monocytes and negative correlations with eosinophils and activated T cells. In MI mice, expression trends of four hub MitoDEGs (*Cox5b*, *Ndufa2*, *Ndufs6*, and *Uqcr11*) were consistent with the bioinformatics results, and their downregulation was associated with reduced cardiac function. CD86 and apoptosis-related proteins (BAX/BCL-2 and cleaved caspase-3) were markedly elevated in MI groups.

**Conclusion:**

These findings suggest that *Cox5b*, *Ndufa2*, *Ndufs6*, and *Uqcr11* act as core regulatory molecules in immunometabolism during MI, providing new insights into its pathogenesis and diagnosis.

## 1 Introduction

Myocardial infarction (MI) is a severe cardiovascular event resulting from prolonged ischemic heart disease, characterized by cardiomyocyte death due to severely restricted or completely blocked blood flow to the myocardium (1, 2). Globally, MI is a leading cause of death and disability, with a 5-year survival rate of approximately 30%, exerting a substantial impact on both health and the economy (3). Although timely diagnosis and effective treatment (4), such as myocardial reperfusion using primary percutaneous coronary intervention (PPCI) for acute myocardial infarction (AMI), can reduce myocardial injury and enhance prognosis, the reperfusion process may induce “myocardial reperfusion injury” which can contribute up to 50% of the final MI size. As such, mortality and morbidity following AMI still remain steady or continue to rise. While cardiac troponins (cTnI/T) remain the gold standard for diagnosing AMI with high sensitivity and specificity, their utility is largely limited to early detection (< 3–4 h post-onset) and acute management. However, growing evidence underscores the need for complementary biomarkers to address unmet clinical challenges in secondary prevention, including risk stratification for adverse remodeling, identification of therapeutic targets, and prediction of long-term outcomes. Current biomarkers lack sufficient prognostic accuracy for detecting subclinical mitochondrial dysfunction and immune dysregulation, which drive chronic pathological processes after MI. Therefore, it is essential to establish such markers not as replacements for cTnI/T but as adjuncts to improve personalized management strategies for MI.

Recent studies have confirmed that the immune response plays a critical role after post-MI (5), such as activating and promoting the accumulation of major immune cell type in ischemic myocardium and orchestrating the pro-inflammatory response (6) to clear necrotic cell debris from the MI zone. Although certain immune cells are essential for cardiac healing, unbalanced or dysregulated immune responses can exacerbate tissue damage (7). Additionally, recent findings suggest that mitochondria play a key role in the regulating inflammatory responses. However, the interaction between mitochondrial metabolism and immune inflammation in MI pathogenesis remains largely unclear, and warrants further investigation. Given the significant contributions of mitochondria and immune responses to MI onset and progression, this study aimed to explore the role of mitochondrial-related genes and their association with the immune system in MI through integrated bioinformatics analyses.

## 2 Methods

### 2.1 Datasets retrieval and processing

**Figure 1:**
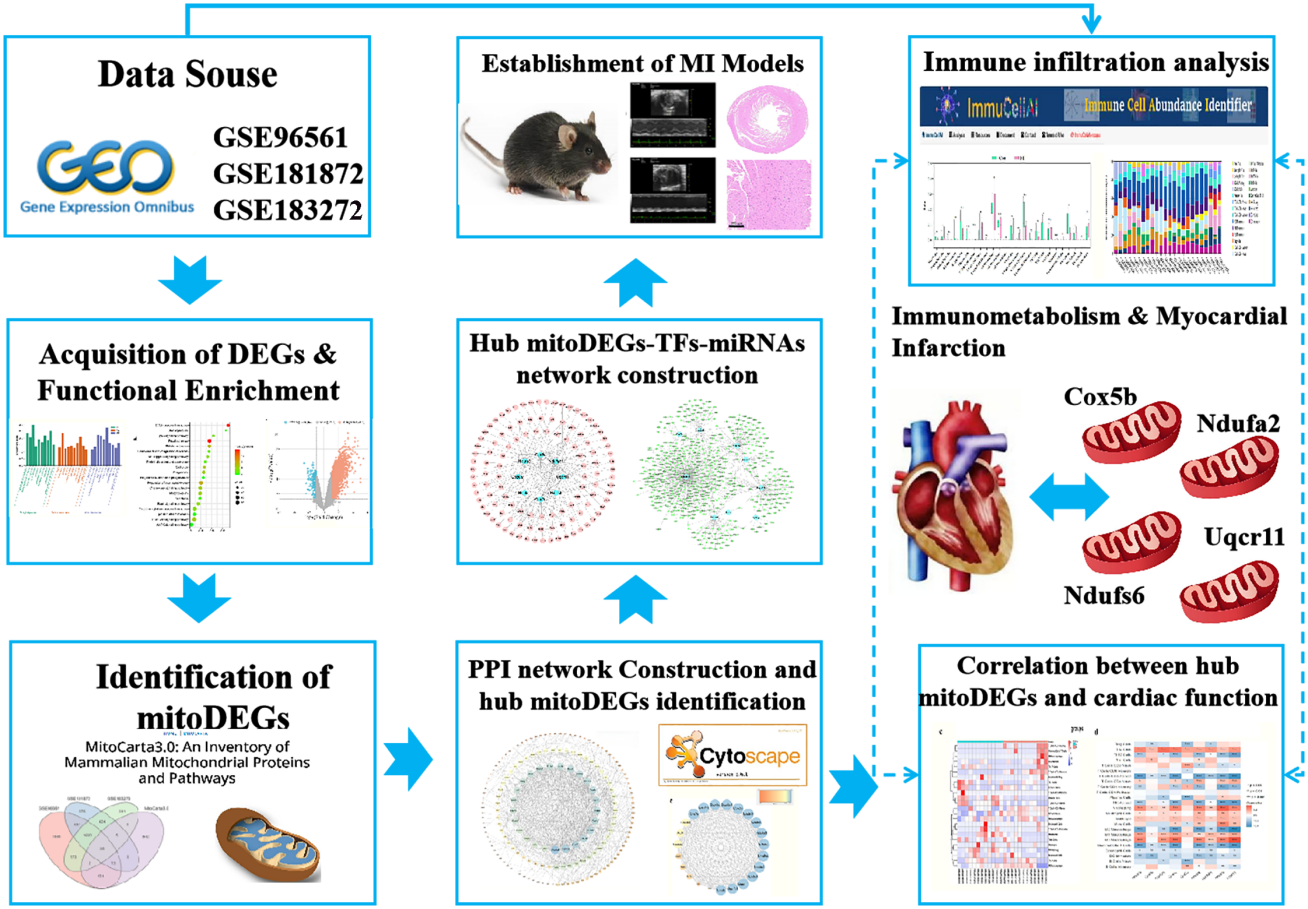
Study workflow on bioinformatics data.

Datasets related to MI were obtained from the Gene Expression Omnibus database (GEO, http://www.ncbi.nlm.nih.gov/geo) using specific criteria: transcriptomics sequencing, *Mus musculus* as the study species, and a model duration of 7 days. Based on these criteria, three datasets-GSE96561, GSE181872 and GSE183272- were selected for further analysis. The GSE96561 dataset, generated using the GLP17021 (Illumina HiSeq 2500) platform and uploaded in 2017, includes transcriptomic data from three MI and three Sham samples. The GSE181872 dataset, generated using the GLP24247 (Illumina NovaSeq 6000) platform and uploaded in 2021, includes eight samples comprising four MI and four Sham samples. The GSE183272 dataset, derived from the Illumina NovaSeq 6000 on the GPL24676 platform, includes five MI and five Sham samples. For integrated analysis, samples from the three datasets were grouped two categories: the MI group (n = 12) and the Sham group (n = 12), as shown in **Table 1**. A simplified workflow is presented in **Figure 1**.

**Table 1:** Descriptive statistics of GEO datasets.

GEO accession	Platform	Sample		Species
		Sham	MI	
GSE96561	GLP17021	3	3	Mus musculus
GSE181872	GLP24247	4	4	Mus musculus
GSE183272	GPL24676	5	5	Mus musculus

### 2.2 Differentially expressed genes analysis and functional enrichment analysis

Standardization, correction, and annotation of gene expression microarray data, as well as identification of DEGs, were performed using the linear models for microarray data (limma) package (version 3.44.0) in R. A conservative threshold (|log2FC| > 0.58, p < 0.05) was applied to identify DEGs between MI and Sham samples. DEG expression patterns were visualized using the “ggplot2” and “ComplexHeatmap” packages in R. Gene Ontology (GO) and Kyoto Encyclopedia of Genes and Genomes (KEGG) enrichment analyses were conducted using the “clusterProfiler” package in R (retaining results with a p < 0.05) and visualized using “ggplot2”.

### 2.3 Identification of mitoDEGs and GO terms analysis

The MitoCarta3.0 mitochondrial protein database (http://www.broadinstitute) was used to extract 1,140 mitochondrial genes (8). MitoDEGs were identified by comparing the DEGs from each microarray with these 1,140 mitochondrial genes using Venn Diagram analysis. Intersecting mitoDEGs across all three microarrays were visualized as a heatmap using the “ggplot2” package in R. Subsequently, GO terms associated with the mitoDEGs were analyzed using the Metascape database (https://metascape.org/) (9).

### 2.4 Construction of protein-protein interaction network and hub mitoDEGs identification

The mitoDEGs were subjected to protein-protein interaction (PPI) analysis using the STRING database (https://string-db.org/). The resulting interaction network was visualized using Cytoscape 3.7.2. Hub mitoDEGs were identified using the Cytohubba plug-in and the molecular complex detection (MCODE) algorithm within Cytoscape 3.7.2.

### 2.5 Hub mitoDEGs-transcription factor -microRNAs network construction

To identify upstream regulators of hub MitoDEGs, the iRegulon plug-in in Cytoscape 3.7.2 was used to predict TFs. Additionally, miRNAs targeting hub mitoDEGs were predicted using the miRWalk database (http://mirwalk.umm.uni-heidelberg.de) (10). A regulatory network comprising hub mitoDEGs, their predicted TFs, and miRNAs was constructed and visualized in Cytoscape 3.7.2.

### 2.6 Immune infiltration analysis

The “limma” package in R was used to standardize gene expression data from GSE96561, GSE181872, and GSE183272 to estimate the proportions of infiltrating immune cells. The standardized data were then analyzed using CIBERSORT (cell-type identification by estimating relative subsets of RNA transcripts), incorporating the LM22 signature and 1,000 permutations. Spearman correlation analysis was used to evaluate the association between hub mitoDEGs and immune cell populations.

### 2.7 Establishment of MI models

Adult male C57BL/6J mice (23–25 g, 9 weeks old) were obtained from SPF (Beijing) Biotechnology Co., Ltd. (certificate number: SCXK [Jing] 2019–0010) and randomly assigned to the Sham and MI groups, with 9 mice in each group. The mice were maintained under controlled environmental conditions at 25 ± 2 °C. All procedures strictly followed laboratory animal care guidelines established by the China Academy of Chinese Medical Sciences (ERCCACMS21-2303-06). MI was induced by permanent ligation of the left anterior descending coronary artery, while Sham group mice underwent the same surgical procedure without ligation. All surgeries were performed under 1.5% isoflurane anesthesia. After 1 week, mice were re-anesthetized, and heart tissues were harvested for further analysis.

### 2.8 Assessment of cardiac function and histopathological evaluation

Transthoracic echocardiography was performed 1 week after MI to assess ventricular geometry and function using the Silicon Wave 30 system (KOLO Medical Co., Ltd). M-mode echocardiography was conducted in the parasternal long-axis view of the left ventricle (LV). Data were averaged from at least three cardiac cycles and included measurements of LV end-systolic posterior wall thickness (LVPWs), end-systolic anterior wall thickness (LVAWs), Ejection fraction and Fractional shortening. H&E and Masson staining were performed according to standard protocols. Heart sections were cut parallel to the short axis and stained with hematoxylin and eosin or Masson’s trichrome. After dehydration and clearing, sections were sealed with neutral gum for imaging.

### 2.9 Quantitative real-time polymerase chain reaction

Total RNA was extracted from heart tissue using TriZol, followed by reverse transcription into cDNA using the HiScript^®^ III RT SuperMix (Vazyme Biotech Co., Ltd). Quantitative real-time polymerase chain reaction (qRT-PCR) was performed using the Taq Pro Universal SYBR qPCR Master Mix kit (Vazyme Biotech Co., Ltd), and amplification was carried out using primers listed in **Table 2**. Target gene expression levels were quantified relative to the glyceraldehyde 3-phosphate dehydrogenase (GAPDH) using the 2^-ΔΔCt^ method.

**Table 2:** Primer sequence of mRNA for qRT-PCR.

No.	GeneName	Sequences
1	*Cox5b*	F: GTCCCGCCCATCTTGCT
		R: GCCAGTGCAATGGCTAATCTTT3
2	*Uqcr11*	F: AAACTGGATTCCCACAGCCG
		R: TGTAAGGCACCCAGTCCAGG
3	*Ndufa2*	F: TTGCGTGAGATTCGCGTTCA
		R: ATTCGCGGATCAGAATGGGC
4	*Ndufs6*	F: GTACGACGCGTGGGGTC
		R: CGACACTTGAACCCCGAAAC
5	*Ndufs3*	F: AGGAACATGGCGGCGGCTGC
		R: ATTTCAGCCACATACTCTCC
6	*Cox6a1*	F: TCAACGTGTTCCTCAAGTCGC
		R: AGGGTATGGTTACCGTCTCCC
7	*Cox7c*	F: CCGTCGCAGCCACTATGAG
		R: TCCAAAGTACACGGTCATCATAGC
8	*Cox6c*	F: GGAGTTGCCGCTGCCTATAA
		R: AATTCTGCATACGCCTTCTTTCTT
9	*Ndufab1*	F: GGACCGAGTTCTGTATGTCTTG
		R: AAACCCAAATTCGTCTTCCATG

### 2.10 Immunohistochemistry

Paraffin-embedded tissue sections were dewaxed, rehydrated, and subjected to antigen retrieval. Endogenous peroxidase activity was blocked using methanol for 15 min. After a 1 h treatment with 5% bovine serum albumin, the sections were sealed and incubated overnight with primary antibodies, including COX5B (dilution 1:200, Abclonal, Inc.), NDUFA2 (dilution 1:200, Abclonal, Inc.), NDUFS6 (dilution 1:200, Proteintech Group, Inc.), and UQCR11 (dilution 1:200, Proteintech Group, Inc.). Sections were then incubated with secondary antibodies for 1 h. Finally, staining was performed using a DAB detection kit, followed by counterstaining with hematoxylin

### 2.11 Western blot

Myocardial tissue samples were homogenized in ice-cold RIPA lysis buffer (P0013B, Beyotime Co., Ltd.) supplemented with protease and phosphatase inhibitors (P002, NCM Biotech Co., Ltd.). Protein concentrations were determined using the BCA assay. Equal amounts of protein (25 µg) were separated by 12% SDS-PAGE and electrotransferred onto PVDF membranes. membranes were blocked with 5% non-fat milk in TBST buffer for 1 h at room temperature. Subsequently, the membranes were incubated overnight at 4°C with primary antibodies against target proteins: anti-COX5B rabbit monoclonal (1:1000, Abclonal), anti-NDUFA2 rabbit polyclonal (1:1000, Abclonal), anti-NDUFS6 mouse monoclonal (1:20000, Proteintech), anti-UQCR11 rabbit polyclonal (1:1000, Proteintech), anti-BCL-2 mouse monoclonal (1:1000, Abclonal), and anti-BAX rabbit monoclonal (1:1000, Abclonal). the membranes were then incubated with HRP-conjugated goat anti-rabbit or goat anti-mouse secondary antibodies (1:10000) for 1.5 h at room temperature. Protein bands were visualized using an ECL chromogenic substrate and quantified using ImageJ software.

### 2.12 Immunofluorescence staining

Tissue sections (3 µm thick) were prepared and stained using a four-color multiplex fluorescence immunohistochemical staining kit (abs50028, Absinbio, Inc.) following the manufacturer’s instructions. After deparaffinization and citrate antigen retrieval, the sections were blocked with 5% goat serum. slides were incubated with the primary antibody anti-F4/80 rabbit monoclonal (1:300, CST) at room temperature for 1 h, followed by incubation with an anti-rabbit horseradish peroxidase-conjugated (HRP) secondary antibody for 10 min and staining with monochromatic fluorescent dye 650 for 10 min. Antigen retrieval was repeated before staining with additional primary antibodies: anti-CD86 rabbit monoclonal (1:300, CST), anti-CD163 rabbit monoclonal (1:500, Abcam), or anti-cleaved caspase-3 polyclonal (1:1000, Absin). Detection was performed using fluorescent dyes 570 and 520 for distinct labeling. Finally, each slide was counterstained with DAPI and mounted with anti-fluorescence quencher sealant. Stained sections were imaged using a Pannoramic MIDI II slide scanner (3DHISTECH).

### 2.13 Correlation between hub mitoDEGs and cardiac function

Correlation analysis between hub mitoDEGs and cardiac function parameters, including LVPWs, LVAWs, Ejection fraction and Fractional shortening, was conducted using the Spearman algorithm and visualized with the “ggplot2” package in R.

### 2.14 Statistical analysis

Bioinformatics analyses were performed using R software (version 4.1.3, https://www.r-project.org/). Statistical analyses were conducted using GraphPad Prism software (version 9.0) with one-way analysis of variance (ANOVA) followed by Tukey’s *post-hoc* test. Statistical significance was defined as p < 0.05, p < 0.01 and p < 0.001.

## 3 Results

### 3.1 Identification of DEGs in MI and functional enrichment analysis

**Figure 2:**
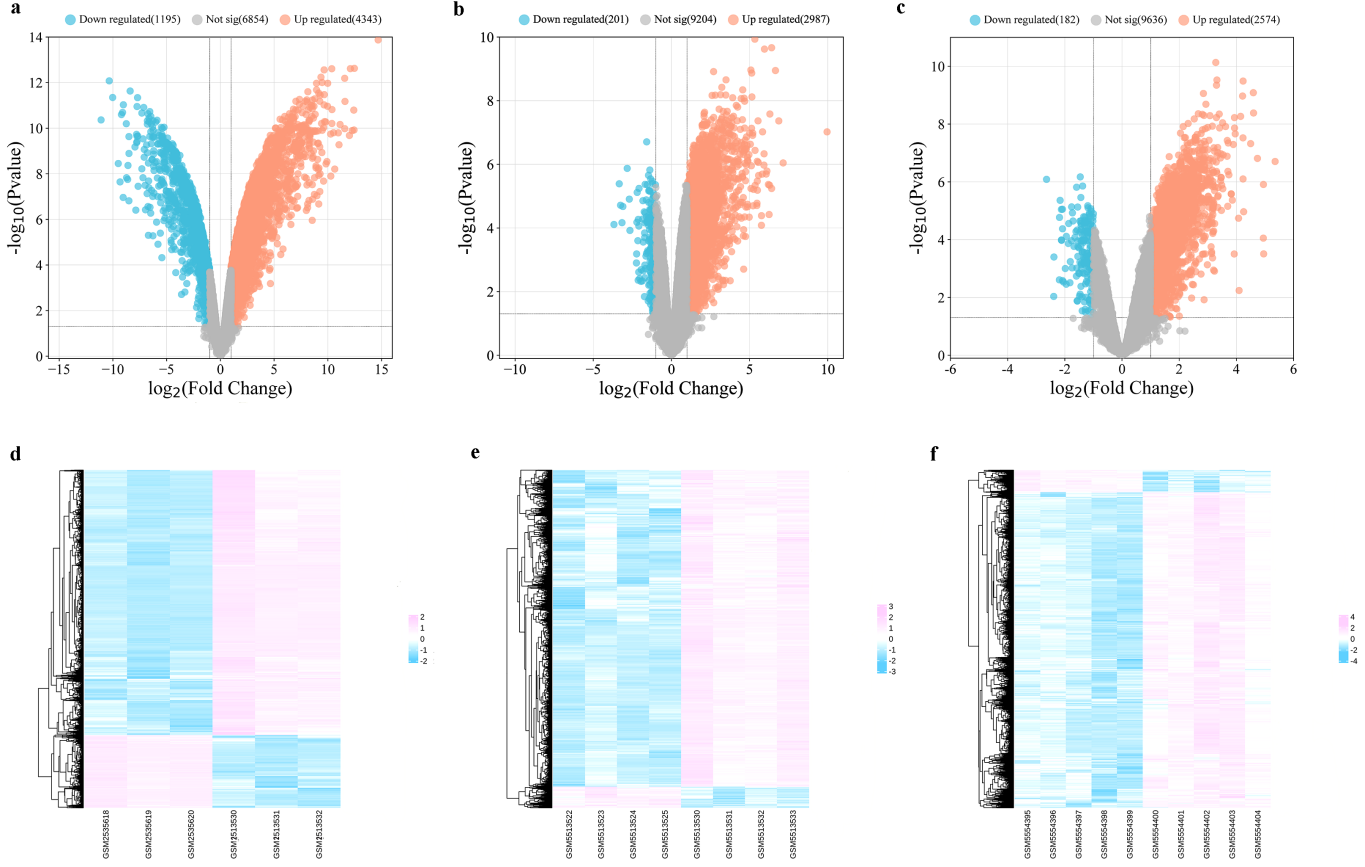
Differentially expressed analysis between MI samples and Sham samples. **(a)** Volcano plot of DEGs in GSE96561; **(b)** Volcano plot of DEGs in GSE181872; **(c)** Volcano plot of DEGs in GSE183272; **(d)** Heatmap of DEGs in GSE96561; **(e)** Heatmap of DEGs in GSE181872; **(f)** Heatmap of DEGs in GSE183272.

Three datasets related to MI (GSE96561, GSE181872, and GSE183272) were obtained for comprehensive analysis. In the GSE96561 dataset, 5538 DEGs were identified, comprising 4343 upregulated and 1195 downregulated genes in MI samples compared to the Sham samples. Analysis of the GSE181872 dataset revealed 3188 DEGs, with 2987 upregulated and 201 downregulated genes in MI samples. In the GSE183272 dataset, 2756 DEGs were identified, including 2574 upregulated and 182 downregulated genes in MI samples compared to the Sham samples. Then, differentially expressed analysis of the integrating GSE96561, GSE181872, and GSE183272 was performed, and the results indicated that there were 5356 upregulated DEGs and 571 downregulated DEGs, which the number of DEGs was fewer than that of separately analysis, and all separately differentially expressed genes analysis contain all DEGs among the integrating GSE96561, GSE181872, and GSE183272, suggesting the results of separately differentially expressed gene analysis of GSE96561, GSE181872, and GSE183272 ensure the accuracy and reliability of the results. Therefore, the separately differentially expressed gene analysis of GSE96561, GSE181872, and GSE183272 were selected for further analysis. As shown in **Figure 2** and **Supplementary Figure 1**, DEGs were visualized using volcano plots and heat maps.

**Figure 3:**
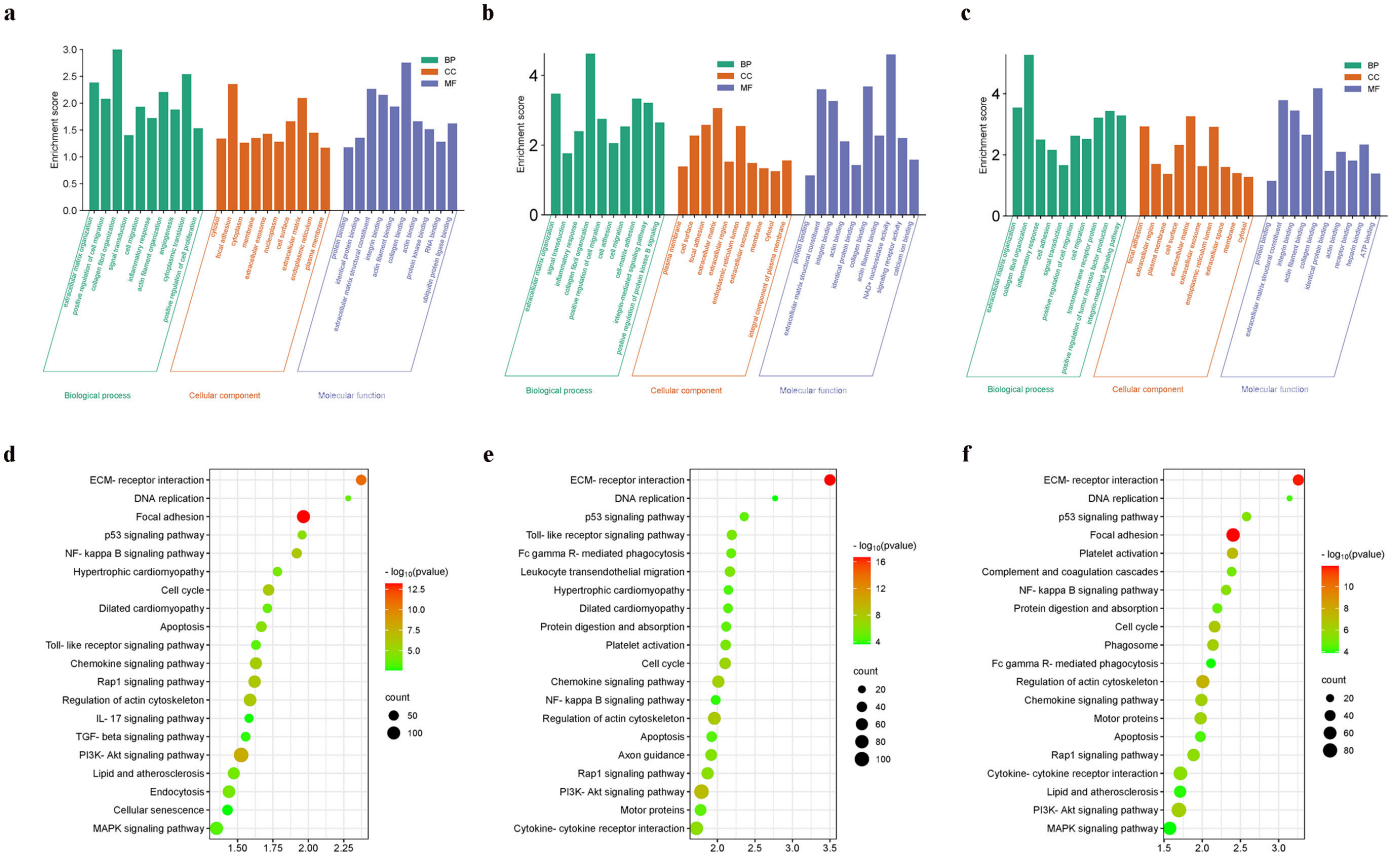
Functional enrichment analysis. **(a)** The enriched GO terms of DEGs in GSE96561; **(b)** The enriched GO terms of DEGs in GSE181872; **(c)** The enriched GO terms of DEGs in GSE183272; **(d)** The enriched KEGG terms of DEGs in GSE96561; **(e)** The enriched KEGG terms of DEGs in GSE181872; **(f)** The enriched KEGG terms of DEGs in GSE183272.

As shown in **Figure 3**, enriched GO terms were categorized into biological processes (BPs), cellular components (CCs), and molecular functions (MFs). Prominent terms included extracellular matrix organization, signal transduction, and binding functions related to the cytosol, membrane, protein, RNA, cell surface, and actin binding. Additionally, KEGG pathway enrichment indicated that the DEGs were primarily involved in the cell cycle, DNA replication, and the PI3K-Akt signaling pathway.

### 3.2 Identification of mitoDEGs in MI and GO terms analysis

**Figure 4:**
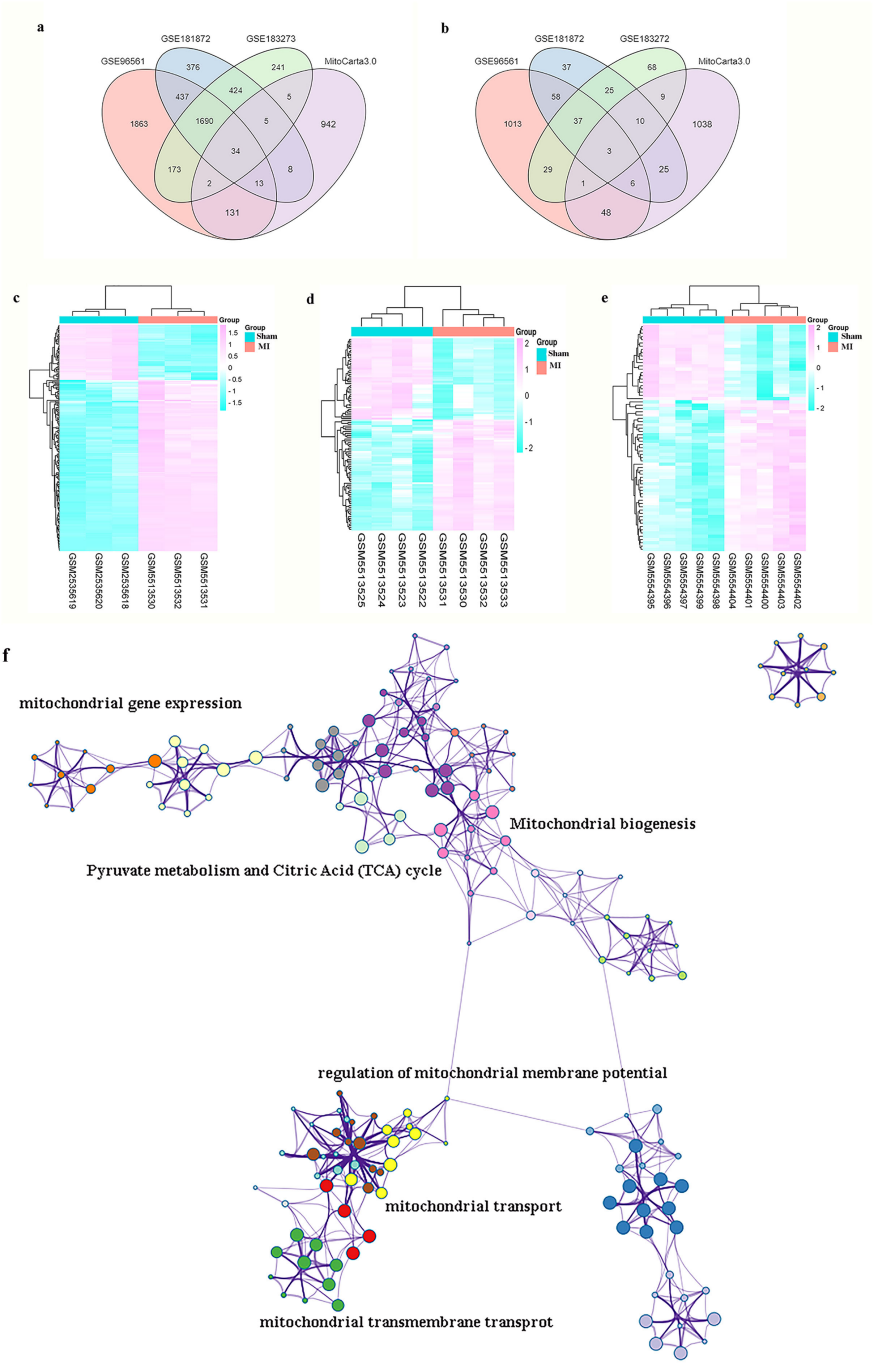
MI and GO terms analysis of mitoDEGs. **(a)** Venn diagrams showed the number of upregulated DEGs between GSE96561, GSE181872, GSE183272 and MitoCarta 3.0; **(b)** Venn diagrams showed the number of downregulated DEGs between GSE96561, GSE181872, GSE183272 and MitoCarta 3.0; **(c)** Heatmap of DEGs both in GSE96561 and MitoCarta 3.0; **(d)** Heatmap of DEGs both in GSE181872 and MitoCarta 3.0; **(e)** Heatmap of DEGs both in GSE183272 and MitoCarta 3.0; **(f)** GO terms analysis of MitoDEGs.

Genes associated with mitochondria were extracted from the MitoCarta3.0 database, and those overlapping with DEGs across the three datasets were defined as mitoDEGs. As shown in **Figures 4a–e**, GSE96561, GSE181872, and GSE183272 contained 238 (180 upregulated and 58 downregulated), 104 (60 upregulated and 44 downregulated), and 69 (46 upregulated and 23 downregulated) mitoDEGs, respectively. Integrating these datasets yielded 294 overlapping mitoDEGs, comprising 196 upregulated and 98 downregulated genes in MI samples compared with Sham samples. These mitoDEGs were primarily enriched in GO terms related to mitochondrial functions, including”mitochondrial gene expression,” “mitochondrial biogenesis,” “pyruvate metabolism and citric acid cycle,” “regulation of mitochondrial membrane potential,” “mitochondrial transport,” and “mitochondrial transmembrane transport” (**Figure 4f**).

### 3.3 PPI network construction and hub MitoDEGs identification

**Figure 5:**
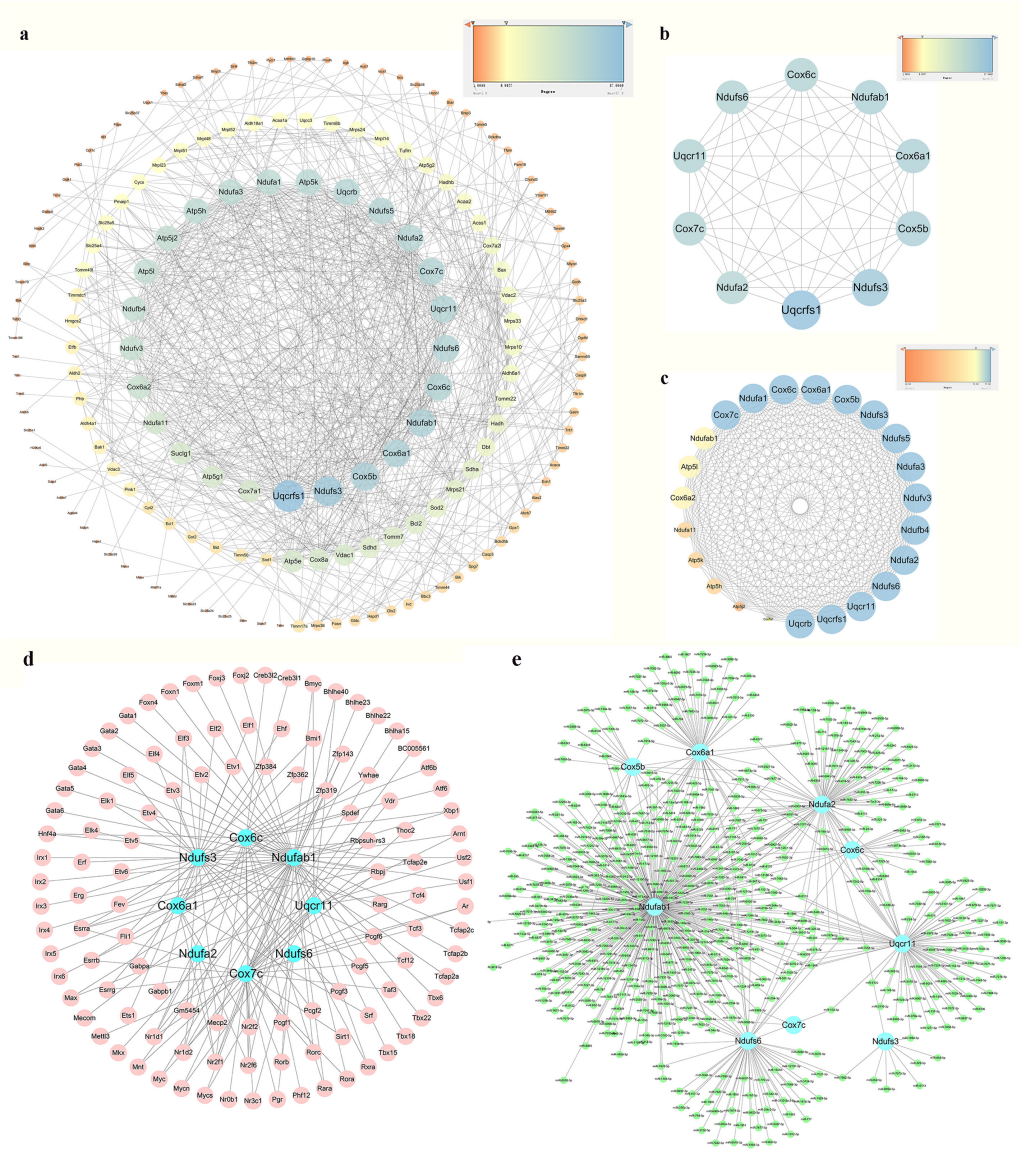
PPI network analysis, hub mitoDEGs identification, and mitoDEGs-TFs-miRNAs regulatory network constructed. **(a)** PPI network of mitoDEGs; **(b)** The top 10 hub genes explored by Cytohubba; **(c)** A significant cluster with 22 genes screened out as hub genes by MCODE; **(d)** TF-hub mitoDEGs regulatory network: the blue dots represent hub mitoDEGs, and the pink dots represent TFs; **(e)** miRNA-hub mitoDEGs regulatory network: the blue dots represent hub mitoDEGs, and the green dots represent miRNAs.

PPI network analysis of the 294 identified mitoDEGs was conducted using the STRING database, applying a combined score threshold > 0.7. The resulting network, consisting of 173 nodes and 777 interaction edges, was visualized in Cytoscape 3.7.2 (**Figure 5a**). Using the degree algorithm of the Cytohubba plug-in and evaluating the network’s topological parameters, 10 candidate hub genes with the highest degrees was identified:*Uqcrfs1*, *Ndufs3*, *Cox5b*, *Cox6a1*, *Ndufab1*, *Cox6c*, *Cox7c*, *Ndufs6*, *Uqcr11* and *Ndufa2* (**Figure 5b**). Gene modules were further analyzed using the MCODE plug-in in Cytoscape 3.7.2 with the following criteria: degree cut-off = 2, node score cut-off = 0.2, k-core = 2, and max depth = 100. As shown in **Figure 5c**, one significant module was identified, comprising 23 nodes and 245 interaction edges, including the genes *Uqcrb*, *Uqcrfs1*, *Uqcr11*, *Ndufs6*, *Ndufb4*, *Ndufv3*, *Ndufa3*, *Ndufs5*, *Ndufs3*, *Cox5b*, *Cox6a1*, *Cox6c*, *Ndufa1*, *Cox7c*, *Ndufab1*, *Atp5l*, *Cox6a2*, *Ndufa11*, *Atp5k*, *Atp5h*, *Atp5j2* and *Cox7a1*. By integrating results from the CytoHubba and MCODE analyses, nine hub mitoDEGs were identified: *Ndufs3*, *Cox5b*, *Cox6a1*, *Cox6c*, *Cox7c*, *Ndufa2*, *Ndufab1*, *Ndufs6* and *Uqcr11*.

### 3.4 Hub mitoDEGs-TFs-miRNAs regulatory network construction

Using the iRegulon plug-in in Cytoscape, TFs associated with hub mitoDEGs were identified, forming a regulatory network composed of 120 nodes (including 9 mitoDEGs and 111 TFs) and 163 interaction edges, as shown in **Figure 5d**. miRNAs associated with hub mitoDEGs were predicted using miRWalk 3.0, yielding a regulatory network with 555 nodes and 656 interaction edges (**Figure 5e**). Within this network, three miRNAs were identified: miR-7075-5p, which interacted with *Ndufab1*, *Cox6a1*, *Ndufa2*, *Ndufs6* and *Uqcr11*; miR-7012-5p, which interacted with *Ndufab1*, *Cox6a1*, *Ndufa2*, *Ndufs6* and *Cox6c*; and miR-6986-5p, which interacted with *Ndufab1*, *Cox6a1*, *Cox5b* and *Ndufs6*.

### 3.5 Immune cell infiltration in MI

**Figure 6:**
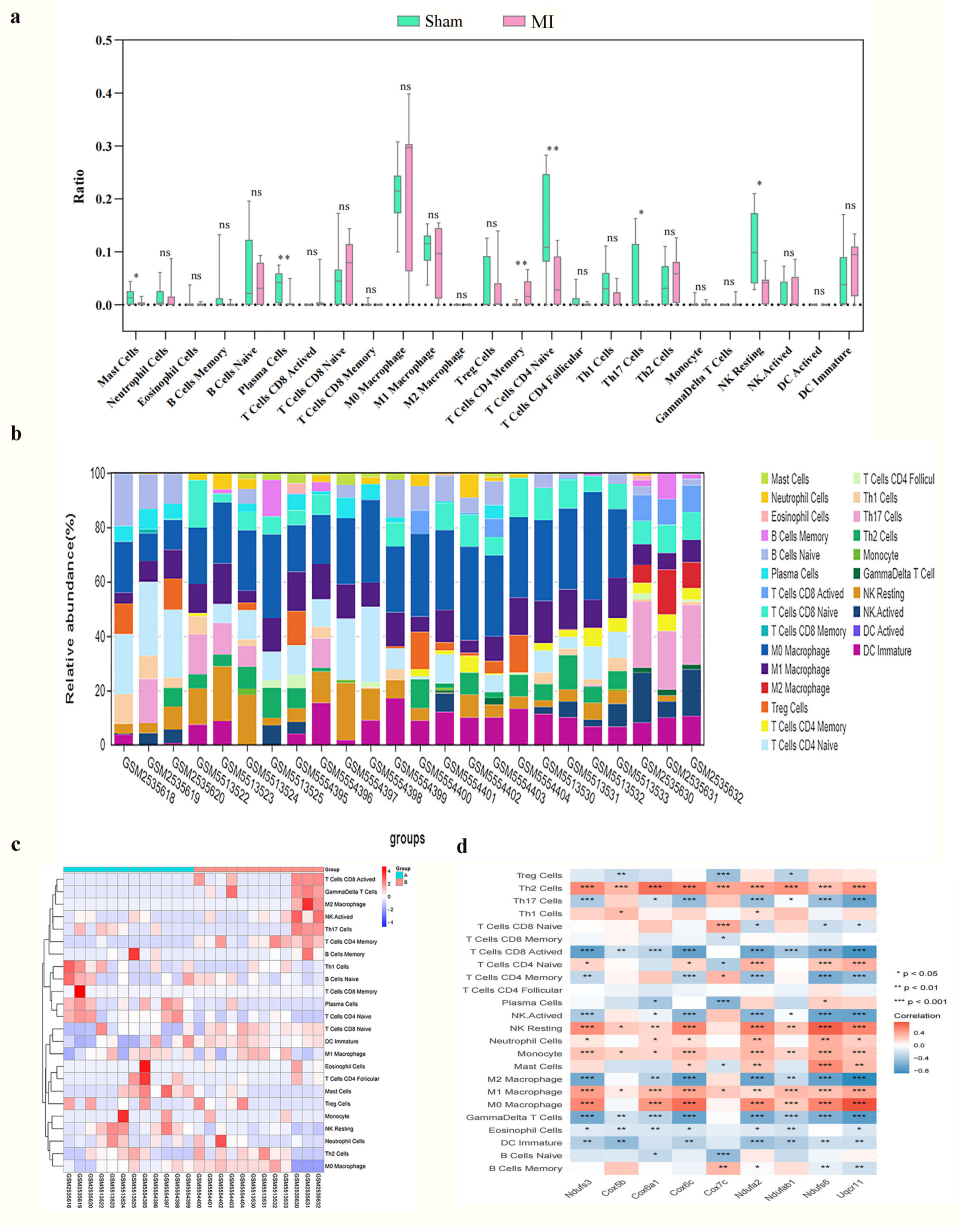
Infiltration of immune cell types compared between the MI and Sham. **(a)** The violin plot of the immune cell proportions; (n=3; *p < 0.05, **p < 0.01 and ***p < 0.001; ns: not significant); **(b)** Stacked bar chart of the immune cell; **(c)** Heatmap of the proportions of 22 immune cell types; **(d)** Correlation between hub mitoDEGs and immune cells.

The CIBERSORT tool was used to analyze the infiltration of 25 immune cell types in the GSE96561, GSE181872, and GSE183272 datasets by comparing MI and Sham samples. Significant differences were observed in the infiltration level of six immune cell types in MI samples (p < 0.05, p < 0.01, respectively). Specifically, mast Cells, plasma cells, Th17 cells, and resting natural killer (NK) cells were more abundant inSham samples, while naïve CD4 T cells were more abundant in MI samples (**Figures 6a, b**). Spearman correlation analysis was then used to assess associations between hub mitoDEGs and immune cell types. As shown in **Figures 6c, d**, *Ndufs3* was positively associated with neutrophils and resting NK cells and negatively associated with eosinophils, activated CD8 T cells, M2 macrophages, CD4 memory T cells, Th17 cells, gamma-delta T cells, and activated NK cells. *Cox5b* showed positive associations with M1 macrophages, Th1 cells, Th2 cells, monocytes, and resting NK cells, and negative associations with eosinophils, activated CD8T cells, Treg cells, gamma-delta T cells, and immature dendritic cells. *Uqcr11* exhibited positively associated with mast cells, neutrophils, M1 macrophages, naive CD4 T cells, Th2 cells, monocytes, and resting NK cells, while negatively associated with eosinophils, memory B cells, activated CD8 T cells, memory CD4 T cells, Th17 cells, gamma-delta T cells, activated NK cells, and immature dendritic cells.

### 3.6 Echocardiography features and pathological staining analysis

**Figure 7:**
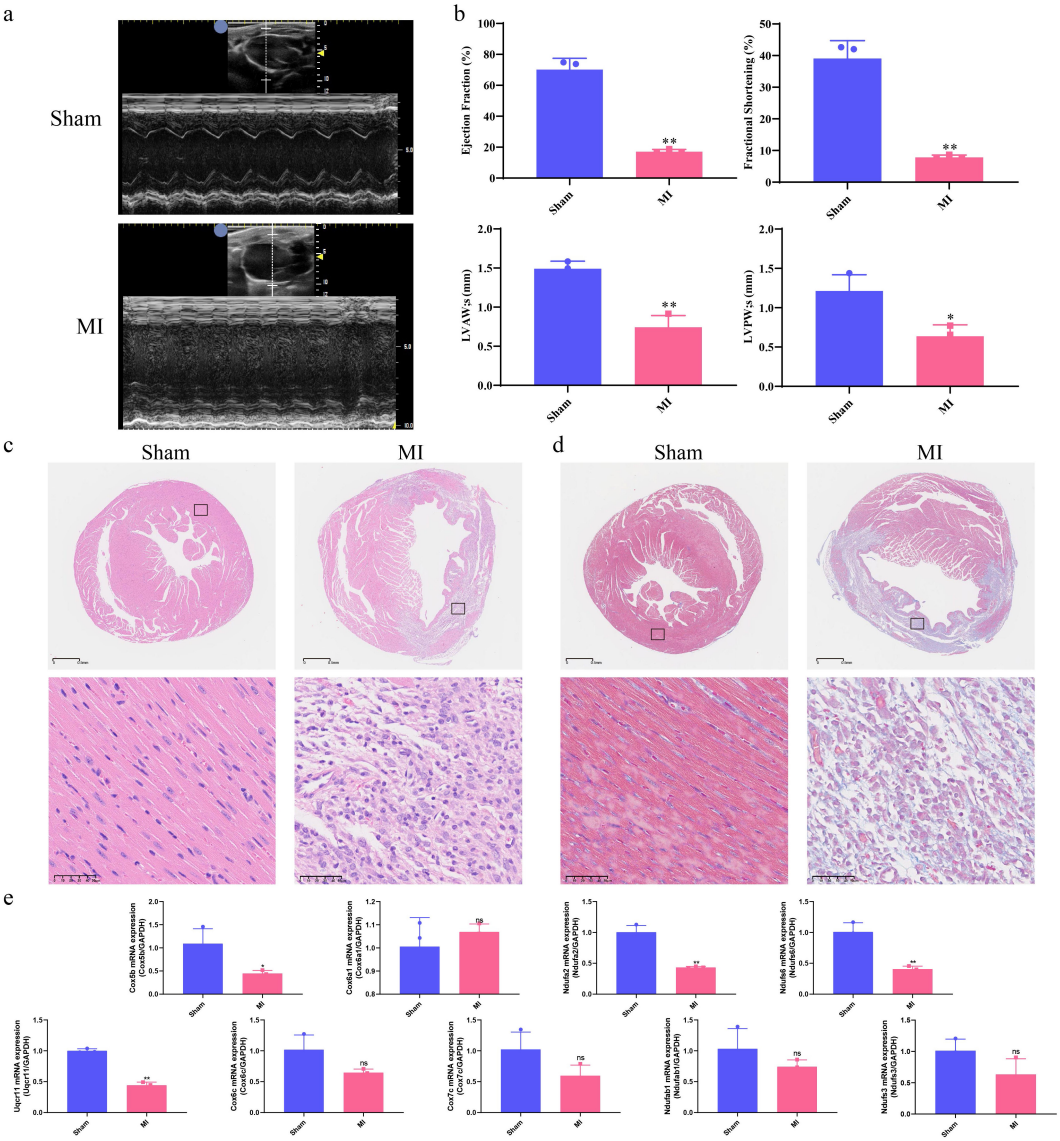
Correlation of hub mitoDEGs expression and association with cardiac function in MI mice. **(a)** Echocardiography of Sham and MI groups; **(b)** Ejection fraction (%), Fractional shortening (%), LVPWs (mm), LVAWs (mm); **(c)** H&E staining of Sham and MI groups (n = 3, bar length: 50 μm); **(d)** Masson’s Trichrome staining of Sham and MI groups (n = 3, bar length: 50 μm); **(e)** Hub mitoDEGs mRNA expression of Sham and MI groups (n=3, *p < 0.05, **p < 0.01, ns: not significant).

As shown in **Figures 7a, b**, echocardiography revealed that the MI group exhibited significantly lower LVPWs (mm), LVAWs (mm), Ejection fraction (%) and Fractional shortening (%) compared to the Sham group (p < 0.05, p < 0.01, respectively). To assess micromorphological heart damage, heart tissues from mice were subjected to H&E and Masson’s trichrome staining. In the Sham group, the cardiac muscle structure showed regularly arranged cardiac fibers and clear myocardial cell striations without pathological alternations. In contrast, the MI group exhibited increased numbers of necrotic, myocardial cells, disorganized fiber alignment, elevated cell death, and greater inflammatory cell infiltration and vacuolization, as shown in **Figures 7c, d**.

### 3.7 Experimental validations of hub mitoDEGs expression and immune cell markers: linking mitochondrial genes to cardiomyocyte survival via immune infiltration and apoptosis-related signaling proteins

**Figure 8:**
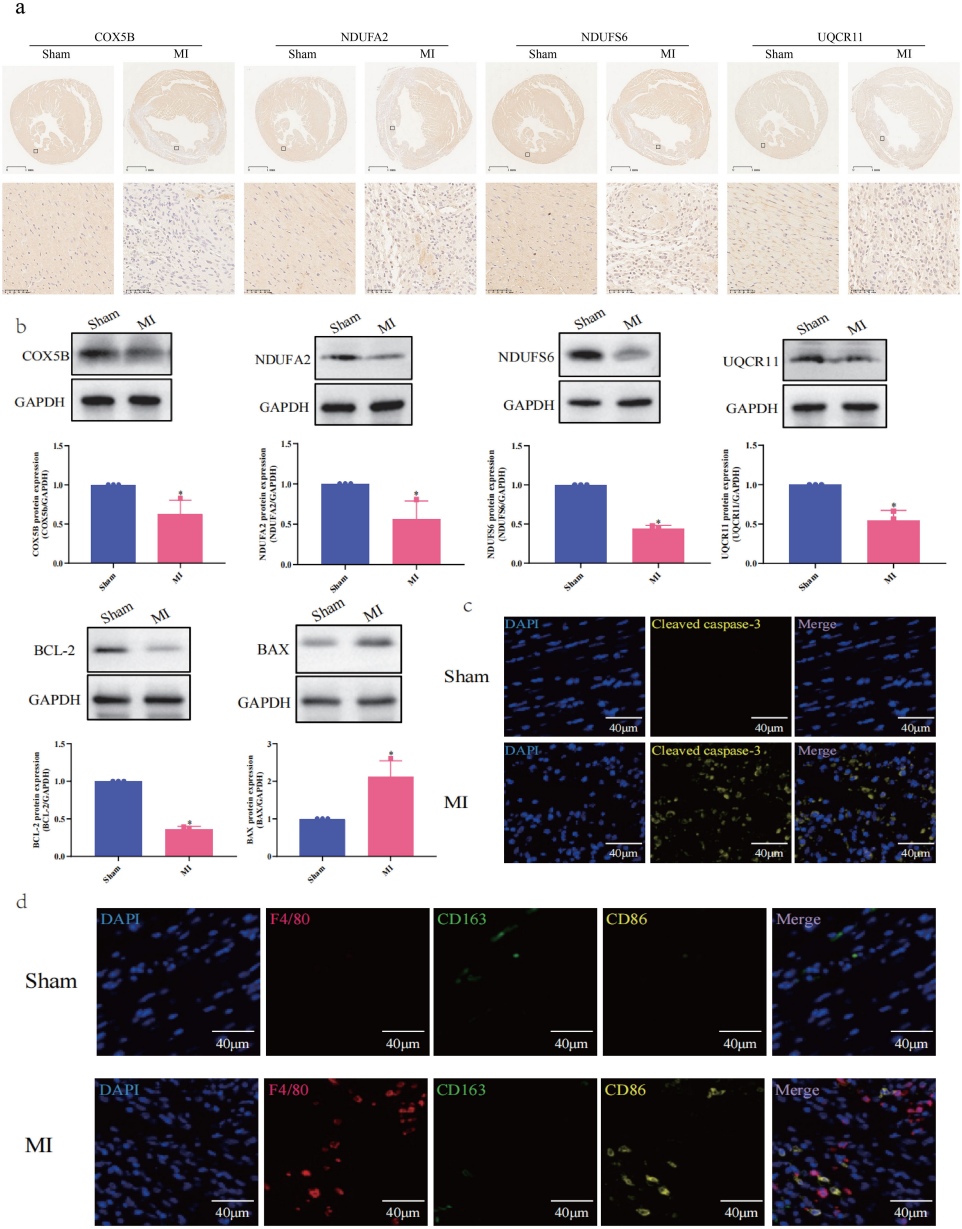
Experimental Validation of Hub MitoDEGs Expression and Immune Cell Markers. **(a)** Immunostaining of protein expression of COX5B, NDUFA2, NDUFS6 and UQCR11; **(b)** Western blotting assay of COX5B, NDUFA2, NDUFS6, UQCR11, BCL-2 and BAX (n=3, *p < 0.05); **(c)** Immunofluorescence Staining for Cleaved caspase-3; **(d)** Immunofluorescence Staining for F4/80, CD163 and CD86.

Ventricular expression of nine hub mitoDEGs (*Ndufs3*, *Cox5b*, *Cox6a1*, *Cox6c*, *Cox7c*, *Ndufa2*, *Ndufab1*, *Ndufs6* and *Uqcr11*) in mice was validated using qRT-PCR. Compared with the Sham group, *Cox5b*, *Ndufa2*, *Ndufs6* and *Uqcr11* showed significantly decreased expression in the MI group (p < 0.05, p < 0.01, respectively, **Figure 7e**). Protein expression of COX5B, NDUFA2, NDUFS6 and UQCR11 were examined separately using Western blot and immunohistochemistry, revealing results consistent with mRNA expression (p < 0.05, **Figures 8a, b**). Western blot and Immunofluorescence staining were subsequently performed to assess apoptosis-related proteins. As shown in **Figure 8b**, BAX expression was markedly increased in the MI group, while BCL-2 expression was reduced. Densitometric analysis normalized to GAPDH revealed a 5.86-fold increase in the BAX/BCL-2 ratio in MI hearts compared with Sham controls (p < 0.05). Immunofluorescence analysis was also conducted to evaluate the expression of cleaved caspase-3, a key executor of apoptosis, in cardiac tissues from MI and Sham groups. As shown in **Figure 8c**, the MI group exhibited significantly elevated cleaved caspase-3 signals at 7 days post-operation compared with the Sham group. Positive staining was primarily observed in cardiomyocytes within the infarct border zone, where apoptotic features such as nuclear condensation and cytoplasmic shrinkage were evident. These findings indicate that MI disrupts the balance between pro- and anti-apoptotic proteins in cardiomyocytes. Subsequently, cardiac immune cell infiltration, particularly of macrophage subsets, was assessed 7 days post-operation using immunofluorescence staining. Macrophages were labeled with the pan-marker F4/80, while M1 and M2 subtypes were identified using CD86 and CD163, respectively. As illustrated in **Figure 8d**, Sham controls showed negligible expression of both CD86 and F4/80 across the myocardium, while CD163 expression was occasionally observed in the anterior left ventricular wall. In contrast, MI mice showed marked accumulation of F4/80^+^ macrophages in the infarcted anterior left ventricular wall. Notably, CD86^+^ M1 macrophages were sparsely detected in this region, while CD163^+^ M2 macrophages remained undetectable across all cardiac sections. These results suggest a predominance of non-polarized macrophages with minimal M1 activation and no discernible M2 response following MI. This spatial distribution pattern highlights the early post-infarct recruitment of non-polarized macrophages, potentially reflecting an incomplete transition between inflammatory and reparative phases.

### 3.8 Relationship between hub mitoDEGs and cardiac function

**Figure 9:**
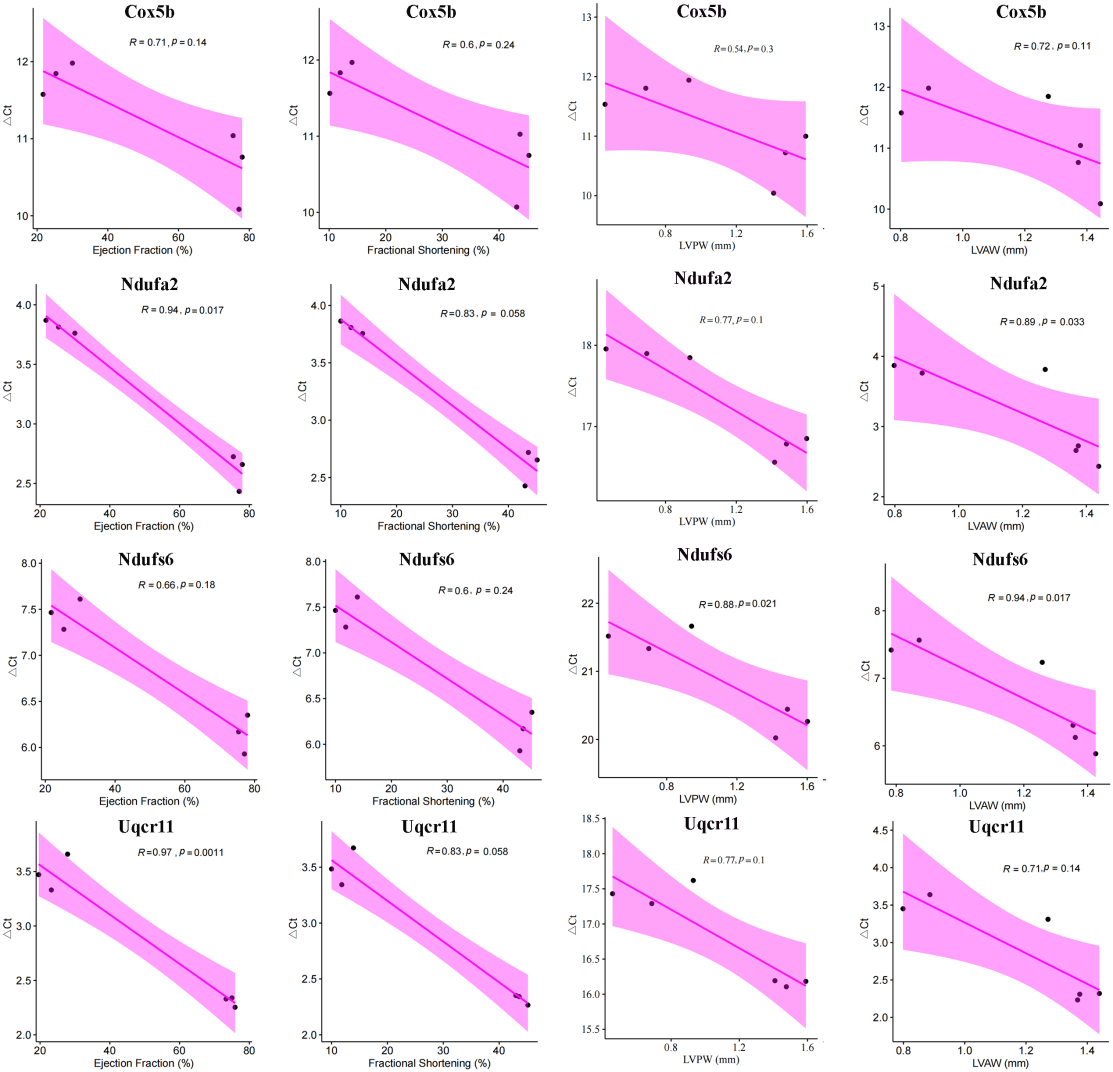
Correlation between hub mitoDEGs and cardiac functional parameters in Sham and MI mice, including Ejection fraction (%), Fractional shortening (%), LVPWs (mm), LVAWs (mm).

Next, the four hub mitoDEGs (*Cox5b*, *Ndufa2*, *Ndufs6* and *Uqcr11*) which exhibited distinct differential expression between the MI and Sham groups were further analyzed for their associations with cardiac function parameters, including Ejection fraction (%), Fractional shortening (%), LVPWs (mm), and LVAWs (mm). As shown in **Figure 9**, PCR cycle numbers for *Ndufa2* were significantly negatively correlated with Ejection fraction (%) (R = 0.94, p = 0.017) and LVAW (mm) (R = 0.89, p = 0.033). PCR cycles of *Ndufs6* showed strong negative correlations with LVAWs (mm) (R = 0.94, p = 0.017) and LVPWs (mm) (R = 0.88, p = 0.021). Similarly, the number of PCR cycles for *Uqcr11* exhibited a strong negative correlation with Ejection fraction (%) (R = 0.97, p = 0.0011). Overall, downregulated expression of *Cox5b*, *Ndufa2*, *Ndufs6* and *Uqcr11* was strongly associated with reduced cardiac function.

## 4 Discussion

MI, characterized by coronary artery obstruction and myocardial necrosis, remains a leading cause of global morbidity and mortality (1). Its pathological cascade involves inflammation, oxidative stress, and apoptosis, which collectively contribute to cardiac dysfunction. Dysfunctional mitochondria have been implicated in various cardiovascular diseases, including MI (11). During MI, an ischemic insult disrupts mitochondrial function, leading to impaired ATP production, elevated reactive oxygen species (ROS) generation, and the release of pro-apoptotic factors, thereby exacerbating myocardial injury. Understanding the role of mitochondria in MI pathogenesis offers promising therapeutic opportunities to preserve mitochondrial function and mitigate myocardial damage. In addition, innate immune cells such as neutrophils and monocytes/macrophages infiltrate the infarcted myocardium during the acute phase to clear cellular debris and initiate tissue repair (11). Based on these findings, this study aimed to elucidate the regulatory roles of mitochondrial metabolism and immune dysregulation in MI progression. Furthermore, this work sought to contribute to a deeper understanding of how mitochondrial metabolism and immune responses intersect in MI pathogenesis and to identify potential therapeutic targets for intervention.

MitoCarta 3.0, a comprehensive mitochondrial proteomic database, was used to identify mitochondrial-associated genes within the GSE96561, GSE181872, and GSE183272 datasets. From these, nine hub mitoDEGs was identified as strongly associated with MI. Additional validation in MI mice models confirmed that four genes, *Cox5b*, *Ndufa2*, *Ndufs6* and *Uqcr11*, exhibited expression patterns consistent with our bioinformatics results. Importantly, the downregulation of *Cox5b*, *Ndufa2*, *Ndufs6* and *Uqcr11* was significantly associated with impaired cardiac function. COX5B, a nuclear-encoded subunit of cytochrome c oxidase (COX), is essential for mitochondrial energy metabolism, functioning in the electron transport chain (ETC) and oxidative phosphorylation (OXPHOS). These findings reinforce growing interest in the role of COX5B in cardiovascular diseases, particularly in the context of MI, which remains a major cause of ischemia-induced cardiomyocyte necrosis and a significant threat to patient survival (12). Emerging evidence suggests that abnormal expression of COX5B is closely associated with the onset and progression of MI. Jarr et al. (13) reported that TNF-like weak inducer of apoptosis (TWEAK) exacerbated left ventricular dysfunction post-MI, with reduced expression of COX5B and NDUFB5 leading to increased cardiac workload through inhibition of PGC-1α. This finding highlights the multifaceted role of COX5B in MI pathophysiology, including its involvement in mitochondrial bioenergetics, oxidative stress response, and cardiac function regulation. NDUFA2 and NDUFS6, both components of mitochondrial respiratory complex I, play essential roles in mitochondrial energy metabolism. NDUFA2 is a nuclear-encoded subunit of complex I, whereas NDUFS6 is a core subunit encoded by mitochondrial DNA. Zhang et al. (14) demonstrated the importance of NDUFA2 in maintaining the stability and function of mitochondrial complex I. Under ischemic conditions, alterations in the expression or activity of NDUFA2 and NDUFS6 disrupt ETC function, resulting in impaired ATP production and increased oxidative stress, thereby exacerbating myocardial injury. UQCR11, a subunit of mitochondrial respiratory complex III, also plays a pivotal role in mitochondrial energy metabolism. As a component of the ubiquinol-cytochrome c reductase complex, UQCR11 facilitates the transfer of electrons from ubiquinol to cytochrome c, a critical step in the ETC and ATP synthesis. This function is vital for maintaining mitochondrial bioenergetics and cellular energy homeostasis. Ma et al. (15) reported that N-Acetyltransferase 10 regulates UQCR11 expression independently of its ac4C enzymatic activity, promoting heart regeneration and suggesting potential therapeutic targets for enhancing myocardial repair.

Mitochondrial dysfunction is a central mediator of cardiomyocyte apoptosis in MI. Our study identified that downregulated hub mitoDEGs (e.g., *Cox5b*, *Ndufa2*, *Ndufs6*, *Uqcr11*) in MI myocardium were associated with disrupted mitochondrial bioenergetics and enhanced apoptotic signaling. Mechanistically, these genes encode subunits of the ETC, and their dysfunction impairs ATP production and mitochondrial membrane potential, thereby triggering cytochrome c release and caspase activation. Western blot analysis revealed a 5.86-fold increase in the pro-apoptotic BAX/BCL-2 ratio in MI hearts, indicating mitochondrial outer membrane permeabilization. Immunofluorescence staining also confirmed cleaved caspase-3 expression in infarcted cardiomyocytes, localized to regions displaying cell shrinkage and nuclear condensation. This caspase-dependent apoptotic cascade was spatially coincident with macrophage infiltration, suggesting a paracrine amplification loop between mitochondrial stress and immune-mediated injury. These findings indicate that MI disrupts the balance between pro- and anti-apoptotic proteins, resulting in enhanced apoptosis and impaired cardiomyocyte survival. This imbalance may be directly linked to the downregulation of mitochondrial genes such as *Cox5b*, *Ndufa2*, *Ndufs6* and *Uqcr11*, which are essential for maintaining mitochondrial function and energy metabolism. The inverse correlation between mitoDEG expression and cardiac function further supports their role in apoptotic regulation and may provide important insights for developing novel therapeutic strategies to improve cardiomyocyte survival and preserve cardiac function.

Metabolic shifts, particularly alterations in mitochondrial metabolism, influence immune cell fate and function (16). During activation, T cells undergo metabolic reprogramming, characterized by increased glycolysis and mitochondrial respiration, to meet the high energy demands associated with proliferation and effector functions (17). In this study, the ImmuCellAI database was used to analyze immune cell infiltration, and higher enrichment of several immune cell types (mast Cells, plasma cells, native CD4 T cells, Th17 cells, and resting NK cells) was found in the Sham group compared to the MI group. Mast cells, recognized for their role in inflammation, have garnered increasing attention due to their involvement in energy metabolism and immune processes (18). These cells release various mediators, such as histamine and cytokines, which influence adipose tissue function and glucose metabolism (19) and have been implicated in the regulation of thermogenesis and energy expenditure, suggesting their involvement in metabolic disorders such as obesity and diabetes (20). Evidence suggests that mast cells accumulate in the infarcted myocardium after MI, where they release inflammatory mediators that contribute to tissue remodeling and fibrosis (21). Hermans et al. reported that mast cell activation is associated with adverse cardiac remodeling, ventricular dysfunction, and poor prognosis in patients with MI, and mast cell-derived cytokines have also been implicated in the pathogenesis of MI-related complications, such as arrhythmias and heart failure (22). Plasma cells are integral components of the immune system and contribute to immunity by secreting immunoglobulins that target pathogens and foreign antigens. Through the secretion of cytokines and chemokines, plasma cells modulate adipocyte function (23) and influence lipid metabolism (24), thereby impacting systemic energy balance. Plasma cell infiltration into the myocardium occurs after MI, contributing to the local immune response and tissue remodeling and exacerbating myocardial injury through the secretion of pro-inflammatory cytokines and the production of autoantibodies targeting cardiac antigens (25). The associations between elevated plasma cell levels or plasma cell-derived antibodies and adverse outcomes in AMI highlights the potential pathogenic role of plasma cells in AMI progression (26). T cells, particularly CD4^+^ T cells, participate in the adaptive immune response by regulating other immune cell activities and modulating immune reactions. Zhou et al. (27) reported that CD4^+^ T cells influence metabolic homeostasis by secreting cytokines that regulate adipocyte function, glucose metabolism, and lipid metabolism and may directly interact with adipocyte to promote their differentiation, contributing to adipose tissue remodeling and metabolic regulation. Gladow et al. (28) explored the impact of CD4^+^ T cells on splenic myelopoiesis and monocyte differentiation following MI and found that conventional CD4^+^ T cells enhance splenic myelopoiesis and promote the pro-inflammatory monocyte differentiation of monocytes, while Tregs mitigate these effects. Th17 cells have been implicated in regulating adipose tissue inflammation (29) and influencing lipid metabolism (30) and glucose homeostasis (31). Emerging evidences suggests that Th17 cells contribute to MI pathophysiology through multiple mechanisms, for instance, the imbalance between Th17 and Treg cells contributes to the initiation and progression of inflammatory and immune responses during allogeneic skeletal myoblast transplantation for AMI (32). Th17 cells have also been detected in the infarcted myocardium following MI, where they exacerbate inflammation and tissue damage by secreting pro-inflammatory cytokines such as interleukin (IL)-6 and IL-23; promoting the recruitment of immune cells, including neutrophils and macrophages, amplifying inflammatory responses, and worsening myocardial injury (33). Clinical investigations suggest that an imbalance between Th17 and Treg cells, with Th17 cell predominance, may serve as a potential therapeutic target and prognostic indicator in patients with cardiogenic shock (34). NK cells, as integral components of the innate immune system, exist in various activation states, including resting and activated states (35). Among these, resting NK cells act as sentinels of the immune system, continuously surveying the body for infected or transformed cells and maintaining metabolic homeostasis through basal energy metabolism processes, including glycolysis and oxidative phosphorylation (36, 37). After MI, the release of damage-associated molecular patterns (DAMPs) from injured cardiac tissues activates resting NK cells, contributing to the elimination of damaged cells and regulating inflammation within the infarcted myocardium (38). In our study, immunohistochemical analysis using macrophage markers (F4/80, CD86, CD163) validated the bioinformatics-predicted immune microenvironment alterations in MI. F4/80^+^ macrophage accumulation in the infarcted myocardium mirrored computational predictions, while sparse CD86^+^ M1 and absent CD163^+^ M2 macrophages indicated impaired polarization. This aligned with the downregulation of hub mitoDEGs (e.g., *Cox5b* and *Uqcr11*) which negatively correlated with M2 macrophages. The absence of CD163^+^ cells suggested an arrested transition to reparative phenotypes, perpetuating inflammation. Spatial overlap of apoptotic cardiomyocytes (cleaved caspase-3^+^) with F4/80^+^ macrophages highlighted a mitochondrial-immune axis: apoptotic debris drives macrophage recruitment, but mitochondrial dysfunction disrupts polarization, exacerbating injury. These findings mechanistically link mitochondrial gene dysregulation to aberrant immune responses, validating macrophage markers as critical nodes in MI pathogenesis and as therapeutic targets for resolving inflammation.

In this study, we explored the interplay between mitochondrial metabolism and the immune microenvironment using comprehensive bioinformatics analyses. We identified four hub MitoDEGs (*Cox5b*, *Ndufa2*, *Ndufs6* and *Uqcr11*) as potential molecular targets for evaluating immunometabolism in MI. Nonetheless, this study had some limitations. The primary limitations include its observational design and the absence of functional validation for the identified hub MitoDEGs. While integrated bioinformatics analysis and experimental validation in murine models provided robust evidence linking mitochondrial dysfunction and immune infiltration in MI, the correlative nature of the findings precludes causal inference. The four hub MitoDEGs (*Cox5b*, *Ndufa2*, *Ndufs6* and *Uqcr11*) remain hypothetical candidates, as their roles in mitochondrial metabolism and immune crosstalk during MI pathogenesis were not mechanistically validated. Specifically, genetic perturbations (e.g., knockout or overexpression) or pharmacological interventions to modulate these genes’ activity, which are critical for establishing causality, were not performed. Additionally, the study relied on murine models and lacked clinical validation, limiting translational relevance. Future studies should prioritize *in vitro* and *in vivo* functional experiments to dissect the roles of these genes in mitochondrial bioenergetics, redox signaling, and immune cell polarization and to explore their therapeutic potential through targeted interventions.

## 5 Conclusion

In conclusion, we identified differences in mitochondrial-related gene expression and immune cell infiltration between MI and Sham groups through multiple bioinformatics analyses and uncovered a crosstalk link between mitochondrial metabolism and immune infiltration in MI. Four hub MitoDEGs (*Cox5b*, *Ndufa2*, *Ndufs6* and *Uqcr11*) identified showed decreased expression in the MI group at both the gene and protein levels, as confirmed by validation experiments. Specifically, *Cox5b*, *Uqcr11*, *Ndufa2* and *Ndufs6* positively correlated with M1 macrophages, Th2 cells, and monocytes and negatively correlated with eosinophils and activated CD8 T cells. These findings suggest that *Cox5b*, *Ndufa2*, *Ndufs6* and *Uqcr11* act as co-regulatory molecules in immunometabolism during MI, offering valuable insights into MI pathogenesis and diagnosis.

## Data Availability

The original contributions presented in the study are included in the article/**Supplementary Material**. Further inquiries can be directed to the corresponding author.

## Supplementary material

Supplementary Figure 1 Differentially expressed analysis between MI samples and Sham samples in the combination of GSE96561, GSE181872 and GSE183272. (a) Volcano plot of DEGs in the combination of GSE96561, GSE181872 and GSE183272; (b) Heatmap of DEGs in the combination of GSE96561, GSE181872 and GSE183272.
