# Supplementary figures and images for: Exploring the role of mitochondrial metabolism and immune infiltration in myocardial infarction: novel insights from bioinformatics and experimental validation
Source: Front Immunol. 2025 May 27;16:1543584. doi: 10.3389/fimmu.2025.1543584 (PMC12148850; doi:10.3389/fimmu.2025.1543584)

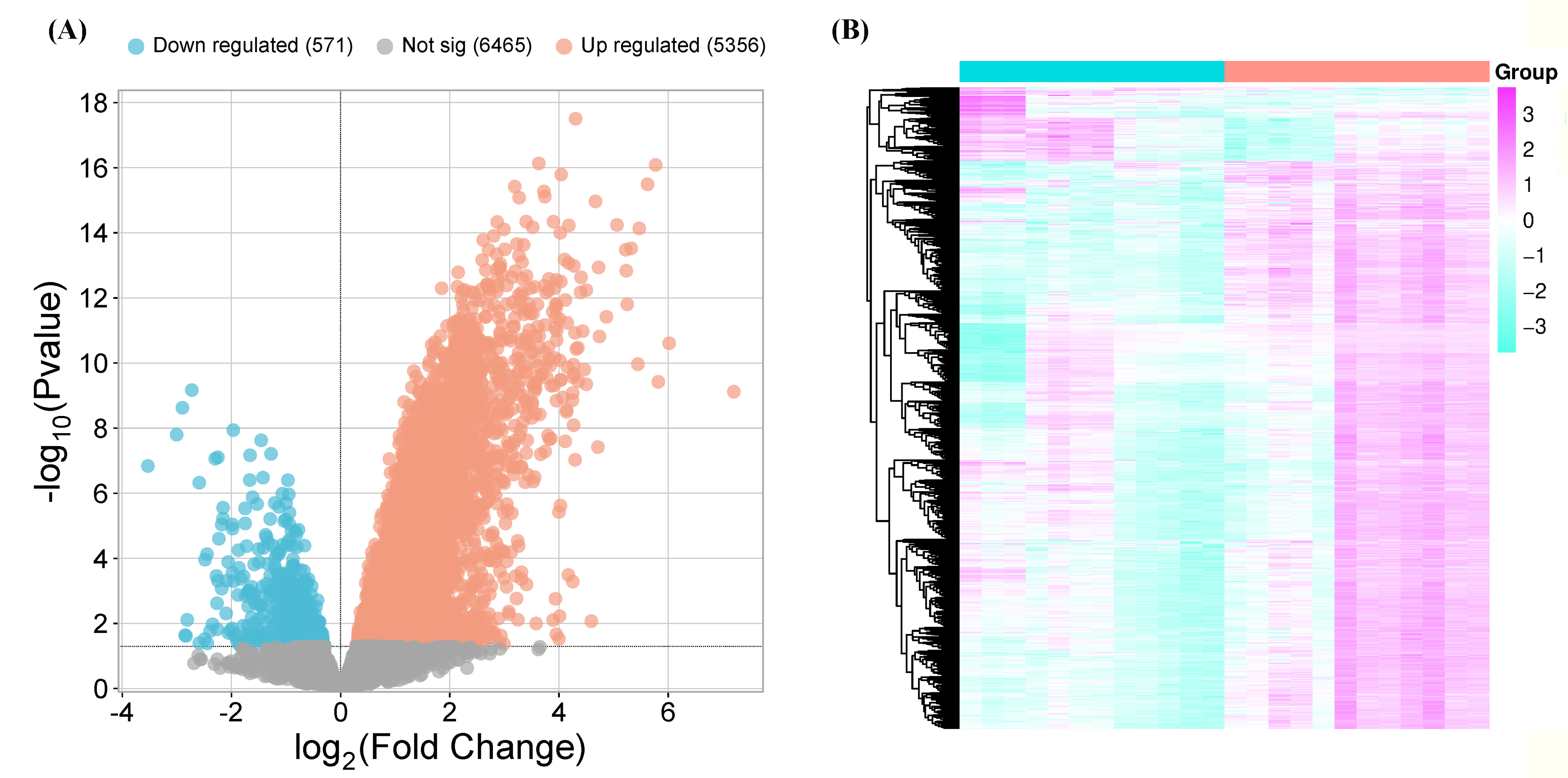

Supplement: Supplementary Figure 1 — Differentially expressed analysis between MI samples and Sham samples in the combination of GSE96561, GSE181872 and GSE183272. (a) Volcano plot of DEGs in the combination of GSE96561, GSE181872 and GSE183272; (b) Heatmap of DEGs in the combination of GSE96561, GSE181872 and GSE183272. [file Image1.tif]
